# Processing and targeting of proteins derived from polyprotein with 2A and LP4/2A as peptide linkers in a maize expression system

**DOI:** 10.1371/journal.pone.0174804

**Published:** 2017-03-30

**Authors:** He Sun, Ni Zhou, Hai Wang, Dafang Huang, Zhihong Lang

**Affiliations:** Biotechnology Research Institute, Chinese Academy of Agricultural Sciences, Beijing, China; Stony Brook University, UNITED STATES

## Abstract

In the transformation of multiple genes, gene fusion is an attractive alternative to other methods, including sexual crossing, re-transformation, and co-transformation, among others. The 2A peptide from the foot-and-mouth disease virus (FMDV) causes the co-translational “cleavage” of polyprotein and operates in a wide variety of eukaryotic cells. LP4, a linker peptide that originates from a natural polyprotein occurring in the seed of *Impatiens balsamina*, can be split between the first and second amino acids in post-translational processing. LP4/2A is a hybrid linker peptide that contains the first nine amino acids of LP4 and 20 amino acids of 2A. The three linkers have been used as a suitable technique to link the expression of genes in some transgenic plants, but to date the cleavage efficiency of three linkers have not been comprehensively demonstrated in the same transformation system, especially in the staple crop. To verify the functions of 2A, LP4, and LP4/2A linker peptides in transgenic maize, six fusion protein vectors that each encoded a single open reading frame (ORF) incorporating two report genes, Green Fluorescent Protein (GFP) and β-glucuronidase (GUS), separated by 2A (or modified 2A), LP4 or LP4/2A were assembled to compare the cleavage efficiency of the three linkers in a maize transient expression system. The results demonstrated the more protein production and higher cleavage splicing efficiency with the polyprotein construct linked by the LP4/2A peptide than those of the polyprotein constructs linked by 2A or LP4 alone. Seven other fusion proteins that each encoded a single ORF incorporating two different genes GFP and Red Fluorecent Protein (RFP) with different signal peptides were assembled to study the subcellular localization of genes linked by LP4/2A. The subcellular localization experiments suggested that both types of signal peptide, co-translational and post-translational, could lead their proteins to the target localization in maize protoplast transformed by LP4/2A polyprotein construct and it implied the LP4/2A linker peptide could alleviate the inhibition of 2A processing by the carboxy-terminal region of upstream protein of 2A when translocated into the ER.

## Introduction

2A is a peptide from the foot-and-mouth disease virus (FMDV), and its co-translational ‘cleavage’ activity is to ‘cleave’ the 2A from the FMDV polyprotein at its own carboxyl terminus. FMDV 2A does not require other domains within the FMDV polyprotein to function and is an autonomous element capable of mediating cleavage [1–5]. This cleavage can occur in a range of heterologous expression systems: viruses [6], insect cells [7], human HTK-143 cells [8], rabbit reticulocytes [9], wheat-germ extracts [10, 11] and HeLa cells [12]. Using 2A for gene fusions has been widely applied in tomato, potato, tobacco, and others [13–15]. In recent years, researchers have attempted to use 2A for multi-gene transformations in staple crops [16, 17]. Ha et al. used 2A to link the phytoene synthase gene from *Capsicum* and the carotene desaturase gene from *Pantoea* to construct a fusion vector that was transformed into rice to generate a high carotenoid content for “Golden Rice” [18]. The application of 2A from the model plant to staple crops indicates that the 2A linker has potential in genetic engineering and biotech breeding for crop improvement. In some cases, however, the cleavage efficiency of shorter versions of 2A may be inhibited by the C-terminus of certain gene sequences upstream of 2A. The use of longer versions of 2A with N-terminal extensions derived from FMDV capsid protein 1D located upstream of 2A (~30 aa in total) was reported to produce higher levels of cleavage [1, 2, 19]. Although the longest F2A sequence (58 aa) was tested to produce the most efficient cleavage, the C-terminal F2A extension of the upstream translation product may have a negative effect on protein confirmation and activity, such as being incapable of correctly producing monoclonal antibodies or expression of enzyme activity. To minimize this effect, a number of laboratories used shorter versions of 2A [8, 20] or incorporated a furin cleavage site between the C-terminus of the upstream protein and the N-terminus of the 2A sequence so that the C-terminal extension was trimmed away. This approach can only be used for secreted proteins because furin is primarily localized within the Golgi apparatus [21].

LP4, a linker peptide originating from a natural polyprotein occurring in seeds of *Impatiens balsamina*, can be split between its first and second amino acids [3, 22]. A chimeric polyprotein composed of two antimicrobial proteins (AMPs) linked by LP4 was cleaved in transgenic *Arabidopsis*, and the proteins were secreted into the extracellular space and exerted antifungal activity *in vitro* [3]. In 2009, Chattoo and Jha used the LP4 linker peptide to connect the antibacterial proteins DmAMP1 and RsAFP2, and they transformed these proteins into rice by *Agrobacterium*-mediated transformation. The transgenic rice exhibited 90% and 79% higher resistance to rice blast fungus and *Rhizoctonia* bacteria, respectively, compared with nontransgenic rice [23]. To avoid the additional sequence residue, a hybrid linker peptide LP4/2A that contained the first nine amino acids of LP4 and the self-processing FMDV 2A was designed and introduced to link the same AMPs, and two individual antimicrobial proteins released from polyprotein precursor. However, the two defensins were directed to different compartments: the first protein were predominantly directed intracellularly whereas the latter protein was secreted [24]. The polyprotein jointed with the LP4/2A linker separates into single proteins with few additional amino acids, and it benefits the proteins functioned correctly in subsequent processes.

This topic has been studied thoroughly for multi-protein sublocalizations, when linked by a 2A-bearing signal peptide in a multi-gene transformation system [25, 26], but not for LP4 or LP4/2A. In mammal cells, both upstream proteins bearing a co-translational signal peptide and downstream proteins with no signal peptide are targeted to the endoplasmic reticulum (ER) [27]. This targeting is most likely because ribosomes are bound to translocons in translation, and downstream protein flow to the ER occurs via translocon, namely, ‘slipstream translocation’. When a downstream protein carried a transmembrane protein signal peptide I (Nonsecretion signal peptide or transmembrane protein signal peptide II), it went to the correct subcompartment. This result was due to the gap between ribosome and translocon, which provided room for the downstream protein to be pushed and correctly led to the target site [12]. Except for the above situations, most proteins are led to their subcompartment by signal peptides [27]. Therefore, signal peptides play a significant role in protein localization in plants and yeast [24, 27].

2A and LP4/2A have worked well in plants; however, the efficiency of 2A cleavage in maize and the effect of the same gene linked by 2A remain unknown. Moreover, the subcellular targeting of the protein linked by LP4/2A was not clear. To assess the efficiency of 2A cleavage in maize and to optimize the 2A system for expression of the same two genes from a bicistronic vector, we generated constructs that encoded two fluorescent reporter proteins, GFP and GUS, in different positions linked by 2A sequences and two GFP linked by various modified 2A versions. To further investigate whether the gene linked by LP4/2A could be correctly localized, GFP and RFP with co-/post-translational signal peptides or simultaneously linked by LP4/2A were constructed. All of the vectors were transformed into maize protoplasts, and a series of assays was completed to answer our questions.

## Materials and methods

### Plasmid construction

All plasmids were constructed using standard methods and were confirmed by nucleotide sequencing (S1 Table).

### Plant materials

The seeds from a maize inbred line (Zong 3) were soaked overnight and then transferred to half vermiculite and half nutrition soil to geminate under light for approximately 3 days until 1.0 cm shoots were visible. The shoots were then moved to the dark chamber (28°C) until the second leaf was approximately 10–15 cm above the first leaf. The plants used for transformation of plasmids containing a chloroplast signal peptide were moved to the light for one day to green the leaf.

### Maize protoplast transformation

The middle part (6–8 cm) of the second leaf was cut into 0.5 mm strips without bruising. The strips were transferred to an enzyme solution (1.5% cellulase R10 (W/V), 0.4% macerozyme R10 (W/V), 0.4 M D-mannitol, 20 mM KCl, 20 mM MES, pH 5.7, 10 mM CaCl_2_, 5 mM β-mercaptoethanol, 0.1% BSA, approximately 0.1 g of leaf in 10 ml of solution) immediately and digested for 6 h at 50 rpm on a shaker in the dark. After digestion, the protoplasts were released thoroughly by shaking at 80 rpm for 2 min, and the enzyme solution containing the protoplasts was filtered with a 100-mesh filter. A protoplast pellet was collected in a round-bottomed tube after spinning at 100 × g for 2 min. The pellet was resuspended in 5 ml of W5 buffer (154 mM of NaCl, 125 mM of CaCl_2,_ 5 mM of KCl, and 2 mM of MES, pH 5.7) and then collected again and kept on ice for 30 min. Before PEG transformation, protoplasts were resuspended in MMg buffer (0.4 M D-mannitol, 15 mM of MgCl_2_, and 4 mM of MES, pH 5.7) at 1–2 × 10^5^ cell/ml. Ten microgram of DNA and 100 μl of protoplasts were added to a 2-ml tube and mixed well, which was followed by adding 110 μl of PEG/Ca^2+^ solution (0.6 g PEG 4000, 750 μl of ddH_2_O, 500 μl of 0.8 M D-mannitol, and 200 μl of 1 M CaCl_2_) and mixing well. The mixture was incubated at room temperature for 20 min, diluted with 440 μl of W5 and mixed well gently at 150 × g for 1 min. Finally, the protoplasts were resuspended in 250 μl of WI (0.5 M D-mannitol, 20 mM of KCl, and 4 mM of MES, pH 5.7) and incubated in 24-well plates in the dark at room temperature overnight.

### GFP fluorometric assay

Following image collection, GFP expression was quantified using the software package Image J (National Institutes of Health, USA). An area of 400 × 300 pixels that contained the highest number of expressing cells was cropped from the series of images and used to quantify GFP [28]. The data were collected from three experiments repeats with three biological replicates of each.

### Confocal laser scanning microscopy

Fluorescent proteins were detected with a Leica TCS SP5 AOBS confocal laser scanning system (400×; Leica Microsystems, Mannheim, Germany). The GFP fluorescence was excited at 488 nm with an Argon laser and was detected between 490 and 540 nm. The chlorophyll fluorescence was detected between 650 and 740 nm, whereas the RFP fluorescence was excited at 543 nm and was detected between 560 and 630 nm. The BFP (Blue Fluorescence Protein) fluorescence was excited at 405 nm with an Argon UV laser and was detected at 410–480 nm.

### GUS histochemical assay

The histochemical analysis of GUS activity was performed as described by Jefferson [29]. The transient expression of the GUS reporter gene was assayed at 24–48 h after the PEG-mediated DNA uptake. The protoplasts were collected by centrifugation at 100 × g for 2 min, and the pellet was resuspended in 0.2 ml of X-gluc solution and then incubated at room temperature for 6–12 h. A drop of protoplast suspension was counted for the number of cells with a microscope. The ratio between the total number of cells plated and the number of GUS-expressing cells was the relative transformation efficiency expressed as a percentage.

### GUS fluorometric assay

The protoplasts were collected by centrifugation at 100 × g for 2 min, and the extraction buffer (0.05 M NaH_2_PO_4_ with pH 7.0, 0.1% SDS, 0.01 M EDTA with pH 8.0, 20% methanol, 0.1% Triton X-100 and 0.1% 2-mercaptoethanol) was added to the protoplasts, which were vortexed to homogeneity. The homogenate was centrifuged at 1,000 × g for 10 min at 4°C, and the supernatant was used for the protein fluorometric quantification assay [30]. The total protein concentration in the extracts was determined by the Bradford method (Protein Assay Kit; Bio-Rad, Hercules, CA, USA). The fluorescence was then measured with excitation at 365 nm and emission at 455 nm on a HITACHI F-4500 spectrofluorimeter. The fluorimeter was calibrated with freshly prepared MU standards in the same buffer. Triplicate assays were conducted in the experiment, and all experiments were repeated three times.

### Immunoblot analyses of GFP, RFP and GUS expression

The transiently transfected maize protoplasts were harvested, mixed with 100 μl of lysis buffer (25 mM of Tris-phosphate with pH 7.8, 1 mM of DTT, 2 mM of DACTAA, 10% (v/v) Glycerol and 1% (v/v) Tween-20), and vortexed. The lysates were then centrifuged for 10 min at 1,000 × g. To detect GFP and RFP, the supernatants, which contained the soluble proteins, were then subjected to 15% SDS-PAGE (approximately 20 μg of soluble protein), and the proteins were transferred to polyvinylidene fluoride (PVDF) membranes. The membrane was blocked using 3% BSA in phosphate buffered saline and 0.1% Tween 20 and was probed using mouse anti-GFP or mouse anti-RFP monoclonal antibodies diluted at 1:2,000 (cwbiotech, China). After three washing steps with PBST, the membrane was incubated with anti-goat antibodies. A Western blotting kit (BioRad) was used to process the immunoblots. To detect GUS or 2A peptide, the supernatants, which contained the soluble proteins, were then subjected to 10% SDS-PAGE (approximately 20 μg of soluble protein), and the proteins were transferred to PVDF membranes. The membrane was blocked using 3% BSA in phosphate buffered saline and 0.1% Tween 20 and was probed using rabbit anti-GUS polyclonal antibodies diluted at 1:5,000 (Abcam, Cambridge, UK) or 2A antibody diluted at 1:2,000. After three washing steps with PBST, the membrane was incubated with anti-goat antibodies. A Western blotting kit (Bio-Rad, Hercules, CA, USA) was used to process the immunoblots.

## Results

### Construction of vectors for the co-expression of two proteins

**Fig 1:**
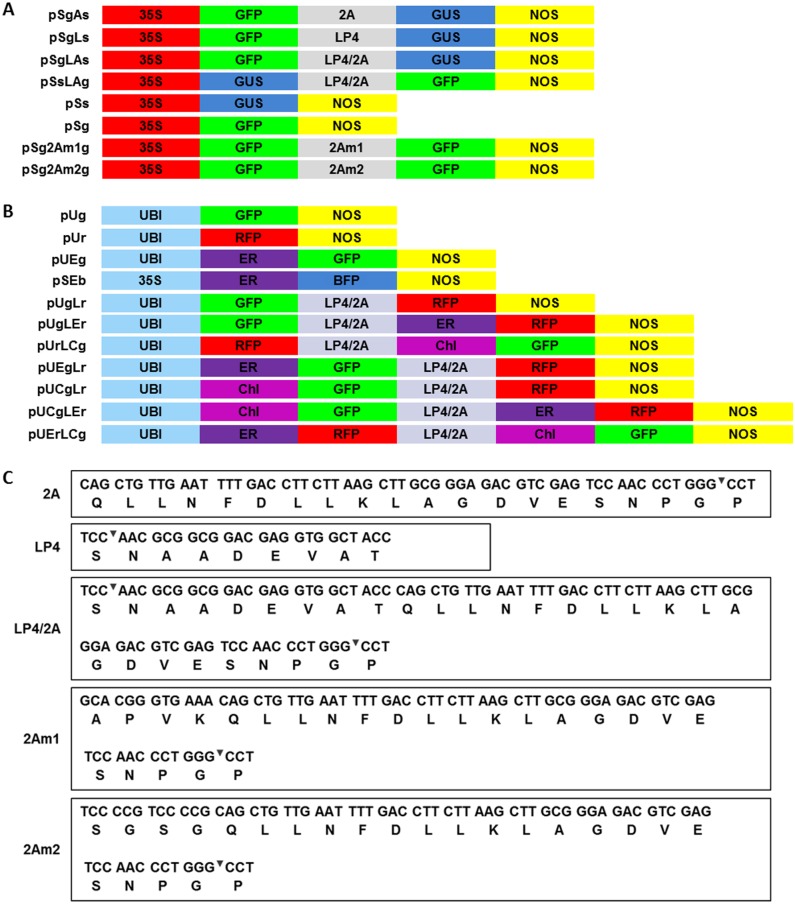
Maps of vectors and sequences of linker peptides. (A) Maps of the vectors used to analyze cleavage efficiency. (B) Maps of the vectors used to analyze subcellular location. (C) DNA and corresponding amino acid sequences of various linker peptides. The underlined sequences encode amino acids APVK and SGSG, which were added to improve cleavage efficiency. 35S: Cauliflower mosaic virus 35S promoter; UBI: Ubiquitin promoter; GFP: green fluorescent protein; RFP: red fluorescent protein; GUS: β-Glucuronidase; NOS: *Nopaline synthase* gene terminator; ER: endoplasmic reticulum signal peptides; Chl: chloroplast signal peptides; 2A, LP4, and LP4/2A: linker peptides.

The constructions shown in Fig 1A were used for cleavage analyses. To compare the efficiency of the different linker peptides, four fusion gene constructs were generated using the two report marker genes GUS and GFP. In the construct pSgAs, 2A was preceded by GFP and followed by GUS. In the construct pSgLs, LP4 was preceded by GFP and followed by GUS. In the construct pSgLAs, LP4/2A was preceded by GFP and followed by GUS. The efficiency of different linker peptides was analyzed with these three constructs. In the construct pSsLAg, the positions of the GUS and GFP genes were exchanged compared with pSgLAs, and the two constructs were analyzed for the effect of gene position. The vectors pSg2Am1g and pSg2Am2g were constructed to ligate two GFP genes with linker 2Am1 and 2Am2, which were longer versions of 2A. The cauliflower mosaic virus 35S promoter was chosen to promote the expression of genes in this series of constructs due to its low activity in maize [31] and to ensure that a slight difference would be easily found.

The construct schematic map shown in Fig 1B was used for gene subcellular location analyses. There were four types of vectors: no signal peptide, signal peptide upstream of linker, signal peptide downstream of linker and signal peptides both upstream and downstream. The other vectors were control vectors. The co-/post-translational signal peptides were designed with different combinations to check their interactions with linker peptides. To realize a strong fluorescent image, maize ubiquitin promoter was chosen for these series of vector constructions. The linker peptide sequences are shown in Fig 1C.

### GUS histochemical assay and fluorometric assay

**Fig 2:**
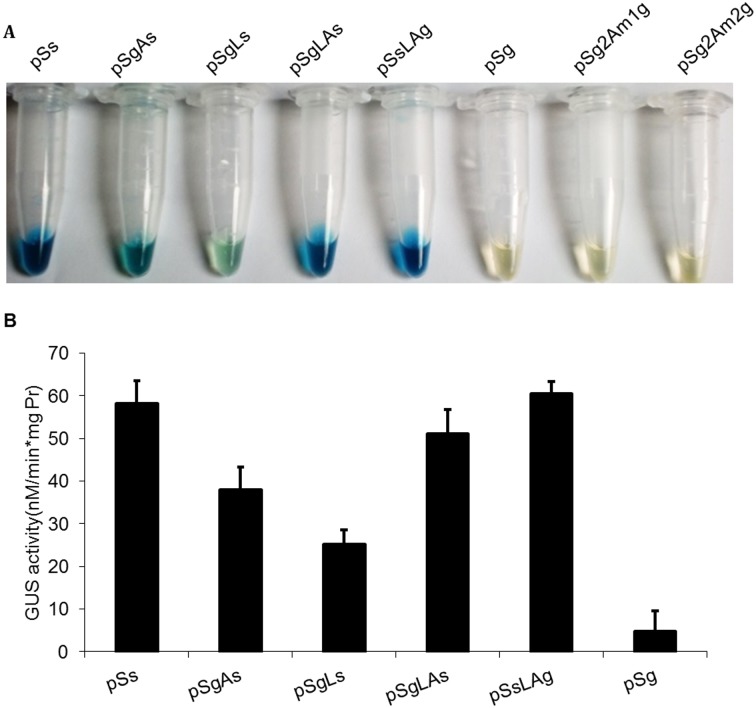
GUS analyses of different vector transformed protoplasts. (A) GUS histochemical assay of different linker peptides in transformed protoplasts. (B) GUS enzyme activity of different linker peptides in transformed protoplasts. The transformed protoplasts were processed for the GUS fluorometric assay 24-h post-transformation. The mean and standard deviation of GUS enzyme activity in each transformed protoplast are presented.

The maize protoplasts harboring pSs, pSgAs, pSgLs, pSgLAs, pSsLAg and pSg were analyzed by the GUS histochemical assay and fluorometric assay to determine the GUS enzyme activity of different vectors. The color depth of the transformed protoplasts shows the enzymatic activity of GUS in Fig 2A. The pSs, pSgLAs and pSsLAg protoplasts were dark blue, whereas the pSgAs and pSgLs protoplasts were light blue. As the negative control, the transformed protoplasts of pSg, pSg2Am1g and pSg2Am2g were transparent and colorless. The quantification of GUS enzyme activity provided additional information that was not easily obtained by visualization (Fig 2B). The activity of GUS in pSsLAg was the same as the activity in pSs but higher than that in pSsAg and pSsLg, which implied that the linker LP4/2A might have more efficient cleavage than 2A and LP4. The activity of GUS in pSgLAs was a little lower than the activity in pSsLAg, 51.08 nM/min*mg protein and 60.49 nM/min*mg protein, respectively. To minimize the transformation efficiency effect, each experiment was independently repeated three times and in each of these three biological repetitions, three technical replicas were made.

### GFP fluorometric assay

**Fig 3:**
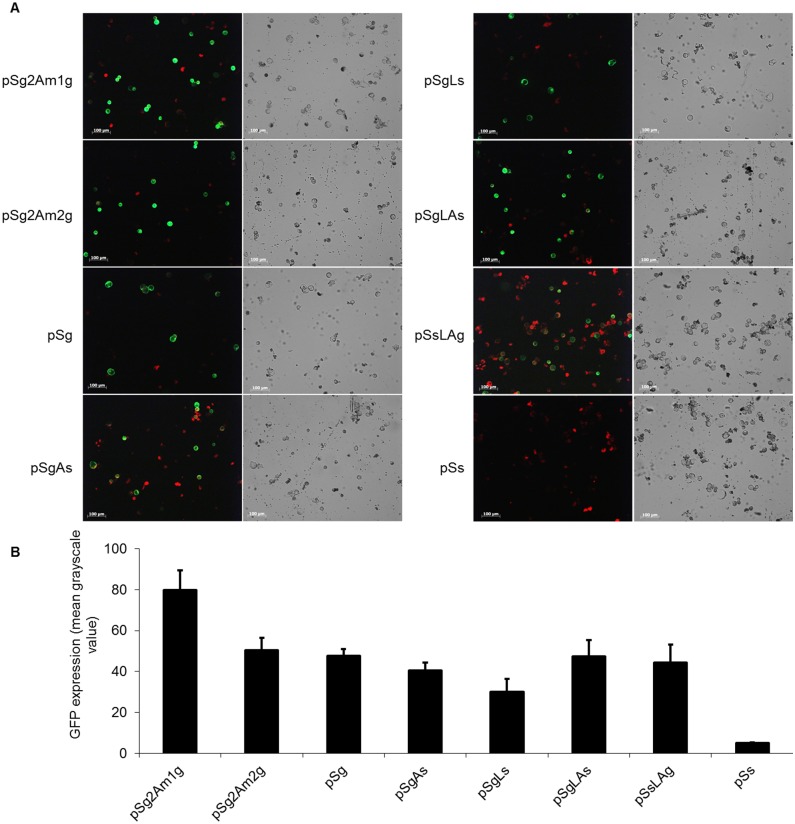
GFP analyses of different vector transformed protoplasts. (A) Confocal microscopy of the transformed protoplasts. Green signals indicate cleaved GFP. The scale bar represents 100 μm. (B) Quantification of GFP expression. The amount GFP expression in each form of vector was estimated from its fluorescence intensity measured by Image J software.

The major differences in GFP expression of different vectors were easily visualized (Fig 3A). The vectors pSg2Am1g and pSg2Am2g exhibited strong GFP fluorescence in transformed protoplasts, and the GFP fluorescence of pSg2Am1g was higher than that of pSg2Am2g. The vectors pSg, pSgLAs, and pSsLAg exhibited moderate strength GFP fluorescence in transformed protoplasts. After image collection, the GFP expression was quantified using Image J. An area of 400 × 300 pixels that contained the highest number of expressing cells was cropped from the series of images and used to quantify GFP. The lowest expression level of GFP was in pSgLs, and the expression level of GFP in pSgAs was also low. Linked by LP4/2A, the expression level of GFP in pSgLAs and pSsLAg was similar to the GFP expression in pSg (Fig 3B). Combined the results of GUS activity in different vectors transformation, the cleavage efficiency LP4/2A was higher than those of 2A and LP4.

### Western blot analysis of GFP and GUS expression in maize protoplasts

**Fig 4:**
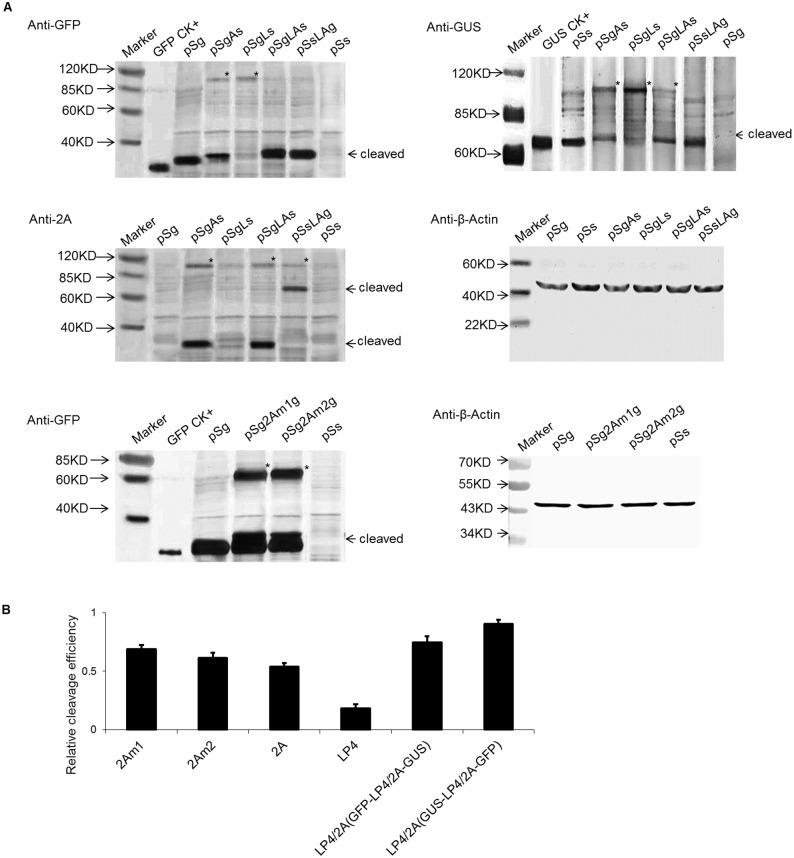
Cleavage efficiency analyses of different vector transformed protoplasts. (A) Western blot analysis of cleavage efficiency of the linker peptide in protoplasts. The transformed protoplasts were processed for Western blot 24-h post-transformation. The cleavage efficiency was assessed using GFP, GUS and 2A antibodies. Anti-β-actin antibody was used as a loading control. Asterisks represent uncleaved proteins. (B) Quantification of the cleavage efficiency of different linker peptides. Cleavage efficiency = cleaved form/(cleaved form + uncleaved form). The amount of each form was estimated from its band intensity on the western blot measured by Image J software.

The protein expression of maize protoplasts harboring pSgAs, pSgLs, pSgLAs, pSsLAg, pSg and pSs was analyzed by Western blotting (Fig 4A) to determine whether the fusion genes were successfully translated and cleaved at the 2A site or the LP4 site to generate monomeric GFP, GFP-2A and GUS. In pSgAs transformed maize cells, the primary band detected by anti-GFP antibodies or 2A antibodies was approximately 30 kDa in size, which was slightly larger than the GFP protein produced in cells expressing pSg. This suggested that the GFP-2A protein cleaved from pSgAs contained the 19-amino acid 2A sequence at its C-terminus. The same extracts were also probed with polyclonal anti-GUS antibodies, and the primary product size detected by the anti-GUS antibodies was nearly 70 kDa, which was the same size as the GUS protein produced in protoplasts expressing pSs. Although the primary products of pSgAs were monomeric GFP-2A and GUS, the uncleaved polyprotein of GFP-2A-GUS was also detected at approximately 90 kDa. Thus, at the level of translation, the efficiency of cleavage of 2A was approximately 60%, as measured by Image J software (Fig 4B). In pSgLs transformed maize protoplasts, the primary band detected by anti-GFP antibodies was approximately 27 kDa in size, which was the same as the GFP protein produced in protoplasts expressing pSg. This suggested that the GFP protein cleaved from pSgLs. The same extracts were also probed with polyclonal anti-GUS antibodies, and the primary product size detected by the anti-GUS antibodies was approximately 71 kDa, which was slightly larger than the GUS protein produced in protoplasts expressing pSs. Although the major products of pSgLs were monomeric GFP and L-GUS, the uncleaved polyprotein of GFP-LP4-GUS was also detected at approximately 90 kDa. Thus, the LP4 worked in maize, and the cleavage efficiency was approximately 30%, as measured by Image J software (Fig 4B). In pSgLAs transformed maize protoplasts, the primary band detected by anti-GFP antibodies or 2A antibodies was approximately 27 kDa, which was the same size as the GFP protein produced in protoplasts expressing pSg. This suggested that the GFP protein cleaved from pSgLAs nearly completely. The same extracts were also probed with polyclonal anti-GUS antibodies, and the primary product size detected by the anti-GUS antibodies was approximately 70 kDa, which was the same size as GUS protein produced in protoplasts expressing pSs. Although the major products of pSgLAs were monomeric GFP and GUS, the uncleaved dimers of pSgLAs and pSsLAg were also detected at approximately 90 kDa. The cleavage efficiency of LP4/2A was approximately 80%, as measured by Image J software (Fig 4B). In pSsLAg transformed maize protoplasts, the primary band detected by anti-GFP antibodies was approximately 27 kDa, indicated that the GFP protein completely cleaved from pSgLAs. The same extracts were also probed with polyclonal anti-GUS antibodies, and the primary product size detected by the anti-GUS antibodies was approximately 70 kDa. The major products of pSsLAg were monomeric GFP and GUS, and no uncleaved 90-kDa products were detected. Thus, the cleavage efficiency for LP4/2A in pSsLAg was approximately 100%, as estimated by Image J software (Fig 4B). Comparing the density of the high- and low-molecular-weight bands, we estimated that the cleavage efficiencies of 2A, LP4, and LP4/2A were approximately 50–60%, 20–30%, and 80–90%, respectively. The results of the GUS antibody and 2A antibody tests were consistent with the results of the GFP antibody test. Compared with GUS 70 kDa size, the 27 kDa GFP is a smaller protein. However, there was no significantly difference in the pSgLAs and pSsLAg and it meant the protein size did not affect the splicing efficiency.

### Localization of processing proteins with co-/post- translational signal peptide in LP4/2A linking polyprotein

**Fig 5:**
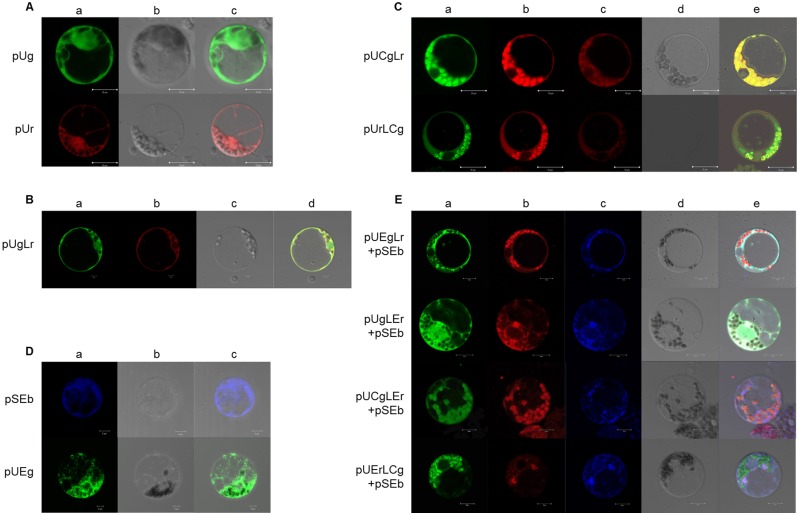
Protoplasts transformed by subcellular locational vectors. (A) Protoplasts transformed by pUg and pUr. A: GFP excitation at 488 nm and RFP excitation at 543 nm; B: light field; and C: AB merge; scale bar = 10 μm. (B) Protoplasts transformed by pUgLr. A: GFP excitation at 488 nm; B: RFP excitation at 543 nm; C: light field; and D: ABC merge; scale bar = 10 μm. (C) Protoplasts transformed by pUCgLr and pUrLCg. A: GFP excitation at 488 nm; B: RFP excitation at 543 nm and emission at 550–710 nm; C: RFP excitation at 543 nm and emission at 550–610 nm (no chlorophyll auto-fluorescence); D: light field; and E: ABD merge; scale bar = 10 μm. (D) Protoplasts transformed by pSEb and pUEg. A: BFP excitation at 405 nm and GFP excitation at 488 nm; B: light field; and C: AB merge; scale bar = 10 μm. (E) Protoplasts transformed by pUEgLr, pUgLEr, pUCgLEr and pUErLCg (co-transformed with pSEb). A: excitation at 488 nm; B: excitation at 543 nm; C: excitation at 543 nm; D: light field; and E: ABCD merge; scale bar = 10 μm.

Some proteins need to function in different subcellular compartments. de Felipe et al [12, 27] have demonstrated that the 2A linked polyprotein with multiple signal sequences could target the correct compartments, and also was found the immediate upstream context of 2A might inhibit the 2A reaction. However, there is no report for LP4/2A linked polyprotein localization so far. To determine whether the linked proteins in the LP4/2A fusion protein system could correctly target the subcellular structure, a series of vectors were constructed with the fluorescence signal and transformed into the maize protoplast. The GFP and RFP in vectors containing only a GFP or RFP gene were primarily localized in the nucleus and cytoplasm (Fig 5A). The fluorescent proteins with no signal peptide were localized in the cytoplasm (Fig 5B).

With an upstream GFP carrying a 141bp Chl (chloroplast) signal peptide from maize rubisco small subunit and a downstream RFP with no signal peptide, the GFP was clearly in chloroplasts, and the RFP was in the cytoplasm, with a small amount of RFP in the chloroplast. With an upstream RFP carrying no signal peptide and a downstream GFP carrying the Chl signal peptide, the RFP appeared in the cytoplasm, and the GFP appeared in the chloroplast; however, the GFP was also detected in the cytoplasm, likely because of minor amounts of uncleaved proteins (Fig 5C). Therefore, proteins with only the Chl signal peptide, whether it was upstream or downstream of LP4/2A, were sublocalized correctly in chloroplasts.

Because it can be difficult to see ER under the microscope, vectors with ER signal peptides were co-transformed together with pSEb (BFP with Calreticulin signal peptide) to maize protoplasts to make a positive control (Fig 5D). When vector had an upstream protein GFP with ER signal and no peptide signal in downstream protein RFP, the GFP was localized in the ER, and the RFP was in the cytoplasm. Additionally, the GFP was localized in the cytoplasm, and the RFP was in ER when the vector had an upstream protein with no ER signal and a downstream protein with an ER signal. When both proteins had a signal peptide, reporter proteins were correctly localized, but minor uncleaved proteins still existed, and the downstream protein followed the upstream protein to co-localize in the same subcompartment (Fig 5E). A summary is presented in Table 1.

**Table 1:** The report proteins’ sublocalization after protoplast transformations with different vectors.

Vector	Upstream protein	Downstream protein	Notes
pUg	Nucleus and cytoplasm		
pUr	Nucleus and cytoplasm		
pUgLr	Cytoplasm	Cytoplasm	
pUCgLr	Chloroplast	Cytoplasm	Minor RFP in chloroplast
pUrLCg	Cytoplasm	Chloroplast	Minor GFP in cytoplasm
pUEgLr	ER	Cytoplasm	
pUgLEr	Cytoplasm	ER	
pUCgLEr	Chloroplast	ER	Minor RFP in chloroplast
pUErLCg	ER	Chloroplast	Minor GFP in ER
pUEg	ER		
pSEb	ER		

### Western blot analysis of GFP and RFP expression

The LP4/2A linker peptide has two cleavage sites; thus, an uncleaved protein would be caused by inadequate cleavage at one or both of the sites. Furthermore, whether the signal peptide was cleaved or not also affected the size of the mature protein. Thus, there would be many possibilities for protein size, as predicted below (Table 2).

**Table 2:** The summary of the prospective proteins and their molecular weights.

Vectors	Uncleavage at LP4 site		Uncleavage at 2A site		Uncleavage at both sites	
	Protein	MW (kDa)	Protein	MW (kDa)	Protein	MW (kDa)
pUgLr	gL+r	29.6+25	g+Lr	26.4+28.2	gLr	55.6
pUCgLr	CgL/gL+r	34.8/29.6+25	Cg/g+Lr	31.6/26.4+28.2	CgLr	60.8
pUEgLr	EgL/gL+r	32/29.6+25	Eg/g+Lr	28.8/26.4+28.2	EgLr	57
pUrLCg	rL+Cg/g	28.2+31.6/26.4	r+LCg/g	25+34.8/26.4	rLCg	59.8
pUgLEr	gL+Er/r	29.6+27.4/25	g+LEr/r	26.4+30.6/25	gLEr	57
pUCgLEr	CgL/gL+Er/r	34.8/29.6+27.4/25	Cg/g+LEr/r	31.6/26.4+30.6/25	CgLEr	62.2
pUErLCg	ErL/rL+Cg/g	30.6/28.2+31.6/26.4	Er/r+LCg/g	30.6/25+34.8/26.4	ErLCg	62.2

MW: Molecular weight; g: GFP protein; L: LP4/2A peptide; r: RFP; C: Chloroplast signal peptide; E: endoplasmic reticulum signal peptide

**Fig 6:**
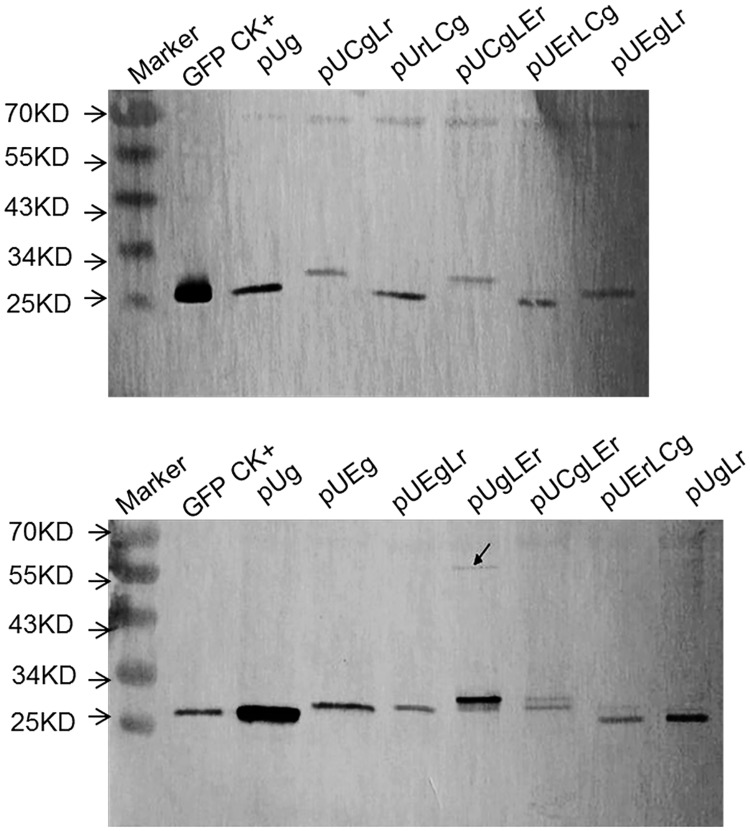
Western blot analysis of report genes expression in subcellular locational vectors. Transformed protoplast was analyzed by western blot to check proteins and their molecular weight, and the antibody is anti-GFP. GFP CK+: GFP positive control; pUg: protoplast transformed by pUg; pUCgLr: protoplast transformed by pUCgLr; pUrLCg: protoplast transformed by pUrLCg; pUCgLEr: protoplast transformed by pUCgLEr; pUErLCg: protoplast transformed by pUErLCg; pUEgLr: protoplast transformed by pUEgLr; pUEg: protoplast transformed by pUEg; pUEgLr: protoplast transformed by pUEgLr; pUgLEr: protoplast transformed by pUgLEr; pUgLr:protoplast transformed by pUgLr.

**Fig 7:**
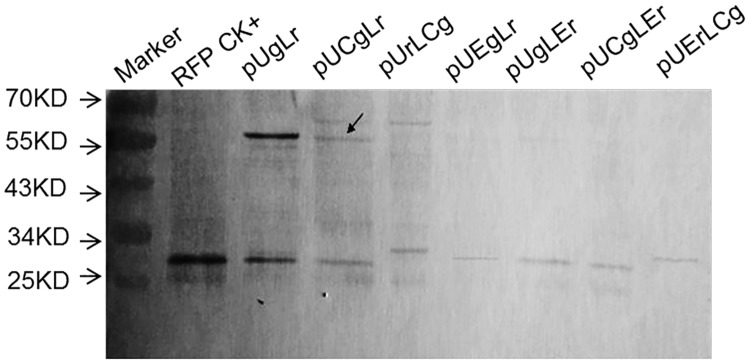
Western blot analysis of RFP expression in transformation protoplast (anti-RFP). Transformed protoplast was analyzed by western blot to check proteins and their molecular weight and the antibody is anti-RFP. RFP CK+: RFP positive control; pUgLr: protoplast transformed by pUgLr; pUCgLr: protoplast transformed by pUCgLr; pUrLCg: protoplast transformed by pUrLCg; pUEgLr: protoplast transformed by pUEgLr; pUgLEr: protoplast transformed by pUgLEr; pUCgLEr: protoplast transformed by pUCgLEr; pUErLCg: protoplast transformed by pUErLCg.

The Western blot probed by GFP antibody showed that every lane had a band approximately 26 kDa, indicating GFP protein or GFP-LP4/2A protein, whereas constructions with LP4/2A also had a weak large band at approximately 60 kDa, indicating uncleaved proteins (Fig 6). The vectors pUCgLr, pUCgLEr, pUEgLr, and pUgLEr were detected at a band larger than 26 kDa respectively, representing a GFPLP42A protein, which meant that the cleavage site of LP4 was not operating (Fig 6). Similarly, the vectors pUrLCg and pUErLCg were detected by RFP antibody with a band of larger than 25 kDa respectively, representing a RFPLP42A, which indicated that the cleavage site of LP4 was malfunctioning as well (Fig 7). Additionally, the proteins at approximately 60 kDa, as indicated by the arrow, were indications of a minor lack of cleavage at both sites (Figs 6 and 7). Neither chloroplast signal peptide nor ER signal inhibited the polyprotein processing, and the cleaved proteins were dominate in the transformed maize protoplast (Figs 6 and 7). However, the residue of LP4/2A at the upstream protein C-terminus about 28 amino acids should give more concern for gene function or activity effect.

## Discussion

Gene stacking is one of the accomplishments in plant genetic engineering in which linker peptides have overcome the drawbacks of traditional methods to some extent and provided a new way to construct gene-stacking vectors. In this study, we compared the cleavage efficiency of five different linker peptides and analyzed the subcellular localization of genes linked by LP4/2A. The results will be a reference for multi-gene transformation research.

### LP4/2A has the highest cleavage efficiency of the five peptide linkers

Although 2A-mediated cleavage might occur in all eukaryotic cells, the use of 2A in maize has not been investigated. In this report, we constructed five vectors with five different linker forms that were transformed into maize protoplast to analyze the gene expression. The two report genes, GUS and GFP, were further measured with a histochemical/fluorometric assay and the Western blot method, respectively. The two detection methods confirmed that the GUS enzymatic activity and GFP expression level in maize protoplasts transformed with pSgLAs were higher than those with pSgAs and pSgLs, which indicated that the LP4/2A peptide was more effective than the 2A peptide or LP4 peptide. We concluded that genes linked by the LP4/2A peptide were more expressed than those linked by the 2A or LP4 peptide alone. However, the cleavage was not complete due to the cleavage site (N-terminus between the S and N and C-terminus between the G and P) of the linker peptide not being completely cut. A prior study of the 2A system concluded that not all of the 2A site could be cleaved completely and that the cleavage efficiency of 2A was different depending on the conditions. Ryan and Drew constructed the vector pCAT2AGUS, which was transformed into rabbit reticulocyte lysates and human HTK-143 cells, and found that 20% of the protein was uncleaved [CAT2AGUS]. The other 80% of the CAT proteins were in the form of CAT with a C-terminal extension of FMDV 2A (CAT2A) and the GUS cleavage product (with an additional N-terminal proline residue), and the products were active [8]. Although LP4/2A linker has been successfully applied in dicot plants genetic engineering [32, 33], few relevant reports about cleavage efficiency were available on LP4/2A. LP4/2A was the linker peptide that combined the first nine amino acids of the LP4 and 2A sequences. Theoretically, this linker peptide has two cleavage sites: one is between the first two amino acids S and N in the N-terminal, and the other is between the last two amino acids G and P in the C-terminal (Fig 1C), which would make the upstream and downstream proteins carry only one amino acid respectively; thus, it would not only minimize the impact of the protein activity or localization but also alleviate the concern for biosafety in transgenic research [24]. In this study, when the polyprotein linked by LP4/2A was cleaved in the translational process, there was also a small amount of fusion protein, meaning the two sites were not cut simultaneously. In Fig 4A, if the protein from the pSgLAs transformation was probed by 2A antibodies, the detected GFP protein was slightly larger than the real GFP, which meant that it was in the GFP-LP4/2A form. When the 2A antibodies probed the protein from the pSsLAg transformation, the GUS-LP4/2A form was detected. This phenomenon implied that the 2A site cleavage efficiency was higher than that of the LP4 site in the LP4/2A linker.

The LP4/2A efficiency in pSsLAg was higher than that in pSgLAs (Fig 4B), which indicated a gene location effect, a phenomenon that was also reported in other papers [34]. In 2003, studies at the University of St. Andrews examined the efficiency of 2A using rabbit reticulocyte lysate and yeast systems. The averages of two independent experiments indicated efficiencies of 88%, 82%, and 76% for DNαF-2A-gfp, ppαF-2A-gfp, and ss△αF-2A-gfp, respectively [9]. Recent studies reported that proteins upstream of 2A might affect the protein construction during translation and then determine whether ‘slipstreaming’ was going to occur [12]; therefore, upstream proteins will likely influence the downstream protein and sublocalization. However, such conditions did not occur when using the fluorescent proteins GFP or RFP. Thus, the order in which the genes are expressed within the construct must be considered. The hybrid peptide LP4/2A had both cleavage mechanisms. If one cleavage efficiency system was low for some specific reason, the other cleavage system offered a suitable alternative, and thus, high efficiency of the fusion protein cleavage was ensured. The LP4/2A system had the higher cleavage efficiency than 2A and LP4, however it did not improve the individual protein expression no matter the upsream protein or downstream protein compared with the single gene construction (Figs 2–4). Francois et al [24] demonstrated an enhanced protein production level in plants transformed with polyprotein construct versus plants transformed with a single protein construct. To further confirm the LP4/2A linker’s function in stable system, the transgenic tobacco and transgenic maize harboring the function genes which were insect-resistant Bt gene and glyphosate-tolerant EPSPS gene linked by LP4/2A were simultaneously conducted, and the same results obtained. The Bt gene and EPSPS gene were spliced correctly and the transgenic plants showed the insect resistance and herbicide tolerance [32, 35].

### Linking the same two genes by using the longer version of 2A improves gene expression

In recent research on 2A, suggestions were made to improve the cleavage efficiency. For example, researchers suggested to add the 1D protein sequence of 2A to the 5' end of 2A, to add some flexible amino acid sequences between the 2A sequence and protein, such as glycine—serine—glycine, and to add other protease recognition sites, among other options [12]. To compare the different 2A versions and improve the gene expression, we examined two forms of 2A: one was with the four amino acids of FMDV 1D protein added, and the other was with the four flexibility amino acids added to connect the GFP genes. The results showed that the GFP expression with two copies of GFP in transformed maize was higher than that with one copy of GFP, although the cleavage efficiency was not 100%. The results also showed that the 2A that had the four amino acids of the 1D protein of FMDV was more efficient than the 2A that had four flexibility amino acids, and the cleavage efficiencies of these two versions were 14.99% and 7.49% higher than that of 2A, respectively. Thus, the amino acids of the 1D protein of FMDV were more helpful for the cleavage of 2A, and the length of the 2A peptide was not the only factor that determined the cleavage efficiency. It was an alternative strategy to improve the foreign gene expression in genetic modified organism through combining the two copies gene linked by 2Am version.

### The LP4/2A linked genes with signal peptide can correctly target the subcellular compartment

Many proteins expressed in transgenic plants play their roles in the subcompartments of cells. Thus, it was necessary to study whether the individual gene in the ‘stacking genes’ targeted the subcellular location. Currently, the stacking gene system with 2A is comparatively well studied, and in most situations, proteins with a signal peptide are able to localize correctly. However, there is also ‘slipstreaming’, when a downstream protein slips into the subcompartment of the upstream protein [36]. In this report, two types of signal peptide, co-translational and post-translational, were used to determine whether there were any differences, and we found that both signal peptides led their proteins to the target localization, whereas ‘slipstreaming’ occurred as well. Considering the possible effects of LP4/2A, three situations could occur without cleavage: an uncleaved LP4 site, an uncleaved 2A site or both sites uncleaved. In this study, the uncleavage primarily occurred at the LP4 site. Efficient cleavage is the foundation of accurate sublocalization of multiproteins in such a gene-stacking system. If the cleavage is inadequate, the downstream protein will likely slip into the subcompartment of the upstream one and will not perform its function. Furthermore, those uncleaved amino acids will interfere with the activity and localization of the mature proteins. Based on our study, most proteins with LP4/2A as a linker, whether with a co-translational signal peptide or a post-translational signal peptide and whether located upstream or downstream of the linker, can be cleaved, with only a few fusion proteins remaining.

The recent research indicated using the signal peptide targeting to the exocytic pathway in the immediate upstream protein of 2A may strongly inhibit the efficiency of 2A reaction, and the authors suggested the use of longer versions of 2A to overcome the inhibition of 2A reaction [12]. The 29aa LP4/2A might inflect the location of 2A and upstream sequence within the ribosome exit tunnel and translocon pore, thus the 2A cleavage function is improved. In this study, the reporter genes linked by the LP4/2A targeted correctly in subcellular compartment and the cleavage efficiency was higher than 2A linker.

Identifying a highly efficient linker peptide and using it to improve gene expression would contribute to the enhancement of multi-gene transformation. In this report, we attempted to find a system that could simplify the process of multi-gene transformation and shorten the time required to obtain multi-gene transgenic material, which would lead to potential applications in both basic academic research and genetic engineering breeding.

## Supporting information

S1 Table Primers for constructing vectors. (DOCX)
