# Supplementary material for: Processing and targeting of proteins derived from polyprotein with 2A and LP4/2A as peptide linkers in a maize expression system
Source: PLoS One. 2017 Mar 30;12(3):e0174804. doi: 10.1371/journal.pone.0174804 (PMC5373624; doi:10.1371/journal.pone.0174804)
Supplement: S1 Table — (DOCX) [file pone.0174804.s001.docx]

**Material and methods:**

S1 Construction of the plasmids used in this study.

pSgAs (p35S-gfp-2A-gus-NOS)

A PCR product containing the *gfp* (S65T) sequence and the fore part of 2A was amplified from pSg (p35S-gfp-polyA), which was deposited in the laboratory, using the oligonucleotide primers PASFp and PASRp (Table S1). The PCR product was digested with *Xba*Ⅰand *Hind* Ⅲ and ligated into the similarly restricted enzyme site of pGEM-7z (Promega, WI) to give the intermediate plasmid p7z-P-2af. A PCR product containing the sequence 2Ab-GUS was amplified from pSs (p35S-gus-NOS), which was deposited in the laboratory, using the oligonucleotide primers PASFs and PASRs (Table S1). The PCR product was digested with *Hind* Ⅲ and *Sac*Ⅰand ligated into the similarly digested pGEM-7z to give the intermediate plasmid p7z-2ab-S. The *Hind* Ⅲ and *Sac*Ⅰfragment of plasmid p7z-2ab-S was cloned in the corresponding restriction sites of plasmid p7z-P-2af to yield p7z-PAS. The *Xba*Ⅰ- *Sac*Ⅰfragment of plasmid p7z-PAS was cloned in the corresponding restriction sites of plasmid pSs to yield pSgAs.

pSgLs (p35S-gfp-LP4-gus-NOS)

A PCR product containing the *gfp* (S65T) sequence and LP4 was amplified from pSg by overlap PCR using the oligonucleotide primers PLSFp, PLSR1p and PLSR2p. The PCR product was digested with *Xba*Ⅰand *Bam* HⅠ and cloned in the corresponding restriction sites of plasmid pSs to yield pSgLs.

pSgLAs (p35S-gfp-LP4/2A-gus-NOS)

A PCR product containing the sequence encoding *gfp* (S65T) and the fore part of LP4/2A was amplified from pSg by overlap PCR amplification using the oligonucleotide primers PLASFp, PLASR1p and PLASR2p. The PCR product was digested with *Xba*Ⅰand *Hind* Ⅲ and ligated into the similarly digested pGEM-7z to give the intermediate plasmid p7z-P-lp4/2af. The *Hind* Ⅲ and *Sac*Ⅰfragment of plasmid p7z-2ab-S was cloned in the corresponding restriction sites of plasmid p7z-P-lp4/2af to yield p7z-PLAS. The *Xba* Ⅰ-*Sac*Ⅰfragment of plasmid p7z-PLAS was cloned in the corresponding restriction sites of plasmid pSs to yield pSgLAs.

pSsLAg (p35S-gus-LP4/2A- gfp -NOS)

A PCR product containing the sequence encoding GUS and the fore part of 2A was amplified by overlap PCR from pSs using the oligonucleotide primers SLAPFs, SLAPR1s and SLAPR2s. The PCR product was digested with *Xba*Ⅰand *Hind* Ⅲ and ligated into the similarly digested pGEM-7z to give the intermediate plasmid p7z-S-lp4/2af. A PCR product containing the sequence 2Ab-GFP was amplified from pSg using the oligonucleotide primers SLAPFp and SLAPRp. The PCR product was digested with *Hind* Ⅲ and *Sac* Ⅰand ligated into the similarly digested pGEM-7z to give the intermediate plasmid p7z-2ab-P. The *Hind* Ⅲ and *Sac*Ⅰfragment of plasmid p7z-2ab-P was cloned in the corresponding restriction sites of plasmid p7z-S-lp4/2af to yield p7z-SLAP. The *Xba*Ⅰ- *Sac*Ⅰfragment of plasmid p7z-SLAP was cloned in the corresponding restriction sites of plasmid pSs to yield pSsLAg.

pSg2Am1g (p35S- gfp - 2Am1- gfp -NOS)

A PCR product containing the *gfp* (S65T) sequence and the fore part of 2Am1 was amplified from pSgAs using the oligonucleotide primers pg2Am1gFp and pg2Am1gRp. The PCR product was digested with *Xba*Ⅰand *Hind* Ⅲ and ligated into the similarly digested pSsAg (p35S-gus-2A-gfp-NOS, deposited in the laboratory) to give the final plasmid pSg2Am1g.

pSg2Am2g (p35S- gfp - 2Am2- gfp -NOS)

A PCR product containing the *gfp* (S65T) sequence and the fore part of 2Am2 was amplified from pSgAs using the oligonucleotide primers pg2Am2gFp and pg2Am2gRp. The PCR product was digested with *Xba* Ⅰand *Hind* Ⅲ and ligated into the similarly digested pSsAg to give the final plasmid pSg2Am2g.

pUg (pUbi-gfp-NOS) and pUr (pUbi-rfp-NOS)

PCR products of *gfp* (S65T) and *rfp* (DsRed2) were amplified from plasmid pSg and pTrfp (kindly provided by Professor Jie Zhang, Institute of Plant Protection, Chinese Academy of Agricultural Sciences ) respectively using primers gfp/B+K-FI3, gfp/B+K-RI4 and rfp/B+K-FI5, rfp/B+K-RI6. And they were restricted by *Bam* HⅠ and *Kpn* Ⅰ then ligated to pGEM-T easy (Promega, WI). Restricted fragments of gfp and rfp were ligated to pUmG2 which was deposited in the laboratory to generate pUg and pUr.

pUEg (pUbi-ERgfp) and pSEb (p35S-ERbfp)

Gfp fragment with ER signal peptide (Arabidopsis basic chitinase) [1] was amplified by overlap PCR by primers ERgfp/s+k-FV17, ERgfp/X+K-FII7 and ERgfp/X+K-RII8. After restricted by *Sma*Ⅰand *Kpn*Ⅰ, it was ligated to pGEM-T easy. Then it was ligated to pUmG2/ *Sma*Ⅰ+ *Kpn*Ⅰto generate the control plasmid pUEg. The bfp fragments with ER signal peptide (Calreticulin signal peptide) at 5’- terminal and HDEL at 3’- terminal was commercially synthesized. After restricted by *Xba*Ⅰand *Sac*Ⅰ, it was ligated to pSg to generate pSEb.

pUgLr (pUbi-gfp-LP4/2A-rfp)

Gfp fragment with fore part of LA was amplified by overlap PCR using primers gfpLP/S+HK-FV11, gfpLP/S+HK-FV12 and gfpLP/S+HK-FV12' meanwhile introducing *Sma*Ⅰrestriction site to its 5’- terminal, *Hin*d Ⅲ and *Kpn* Ⅰto 3’- terminal. Rfp fragments with posterior part of LA was amplified by PCR using primers LPrfp/H+K-V13 and LPrfp/H+K-V14 meanwhile introducing *Hin*d Ⅲ restriction site to its 5’- terminal, *Hin*d Ⅲ and *Kpn* Ⅰto 3’- termianl. Above two fragments were ligated to pGEM-T easy and sequenced. Then they were ligated to pUmG2/ *Sma*Ⅰ+ *Kpn*Ⅰto generate pUgLr.

pUCgLr (pUbi-Chlgfp-LP4/2A-rfp) and pUEgLr (pUbi-ERgfp-LP4/2A-rfp)

A 141bp Chloroplast signal peptide (Chl) from maize rubisco small subunit [2] was amplified by PCR from pUmG2 using primers Chl/X+B-FI1 and Chl/X+B-RI2 meanwhile introducing *Xba*Ⅰrestriction site to its 5’-terminal and *Bam* HⅠ to 3’- terminal. Then it was ligated to pGEM-T easy to get pTCh. Gfp fragment restricted by *Bam* HⅠ and *Kpn*Ⅰfrom pTgfp was inserted to pTChl to obtain pTCg. CgL fragment with front part of LA was amplified by overlap PCR from pTCg using primers CHgL/S+H+K-FⅢ15, CHgL/S+H+K-RⅢ16 and CHgL/S+H+K-RⅢ17. Lr fragment restricted by *Hin*d Ⅲ and *Kpn* Ⅰ from pTLr was inserted to pTCg to get pTglr. Finally, glr fragment was inserted to pUmG2/ *Sma*Ⅰ+ *Kpn*Ⅰto generate pUCgLr. Lr fragment from pTLr was inserted to pUCEgL (synthesized by Genscript, Nanjing, China) by restricted *Hin*d Ⅲ and *Kpn*Ⅰto obtain pUCEgLr. And EgLr fragment was inserted to pUmG2/ *Sma*Ⅰ+ *Kpn*Ⅰto generate pUEgLr.

pUrLCg (pUbi-rfp-LP4/2A-Chlgfp) and pUgLEr (pUbi-gfp-LP4/2A-ERrfp)

Restricted by *Bam* HⅠand *Kpn*Ⅰ, gfp fragment of pTg was ligated to pUC7LC (synthesized by Genscript, Nanjing, China) to get pUC7LCg. LCg fragment of pUC7LCg restricted by *Hin*d Ⅲ and *Kpn*Ⅰligated to pUCrL (synthesized by Genscript, Nanjing, China) to get pUCrLCg. Finally, rLCg fragment was inserted to pUmG2/ *Sma*Ⅰ+ *Kpn*Ⅰto generate pUrLCg.

Rfp with ER signal was amplified by overlap PCR from pTrfp using primers ERrfp/X+K-FII9', ERrfp/X+K-FII9 and ERrfp/X+K-RII10. Er fragment with posterior part of LA was amplified from pTEr using primers LERr/H+K-FV19 and ERrfp/X+K-RII10 meanwhile introducing *Hin*d Ⅲ restriction site to its 5’- termianl, and *Kpn*Ⅰ to 3’- termianl. And it was ligated to pGEM-T easy to get pTLEr. LEr fragment was inserted to pTgL to get pTgLEr and then gLEr was inserted to pUmG2/ *Sma*Ⅰ+ *Kpn*Ⅰto generate pUgLEr.

pUCgLEr (pUbi-Chlgfp-LP4/2A-ERrfp) and pUErLCg (pUbi-ERgfp-LP4/2A-Chlgfp)

LEr fragment of pTLEr was restricted by *Hin*d Ⅲ and *Kpn* Ⅰ then ligated to pTCgL to get pTCgLEr. CgLEr fragment was inserted to pUmG2/*Sma*Ⅰ+ *Kpn*Ⅰto generate pUCgLEr. LCg fragment of pUC7LCg was restricted by *Hin*d Ⅲ and *Kpn*Ⅰthen ligated to pUC7ErL to get pUC7ErLCg, then ErLCg was inserted to pUmG2/*Sma*Ⅰ+ *Kpn*Ⅰ to generate pUErLCg.

**Table S1 Primers for constructing vectors**

| Name | Primers sequence | |
| --- | --- | --- |
| PASFp | 5'CTAGTCTAGATAACCATGGTGAGCAAGGGCGAGGAGCTG |  |
| PASRp | 5'CCCAAGCTTAAGAAGGTCAAAATTCAACAGTTGCTTGTACAGCTCGTCCATGC |  |
| PASFs | 5'CCAAGCTTGCGGGAGACGTCGAGTCCAACCCTGGGCCTATGTTACGTCCTGTAGAAAC |  |
| PASRs | 5' GAGCTCTCACACGTGGTGGTGGTGGTGGTGGCTA |  |
| PLSFp | 5'CTAGTCTAGATAACCATGGTGAGCAAGGGCGAGGAGCTG |  |
| PLSR1p | 5'GTCGGCGGCGTTGCTCTTGTACAGCTCGTCCATGC |  |
| PLSR2p | 5'GGATCCTGTTGCCACCTCGTCGGCGGCGTTGCT |  |
| PLASFp | 5'CTAGTCTAGATAACCATGGTGAGCAAGGGCGAGGAGCTG |  |
| PLASR1p | 5'CCCAAGCTTAAGAAGGTCAAAATTCAACAGCTGGGTAGCCACCTCGTCCGCCGC |  |
| PLASR2p | 5' GGTAGCCACCTCGTCCGCCGCGTTGGATTTGTACAGCTCGTCCATGC |  |
| PLASFs | 5'CCAAGCTTGCGGGAGACGTCGAGTCCAACCCTGGGCCTATGTTACGTCCTGTAGAAAC |  |
| PLASRs | 5' GAGCTCTCACACGTGGTGGTGGTGGTGGTGGCTA |  |
| SLAPFs | 5' GTAGTCTAGATAACCATGTTACGTCCTGTAGAAACCCCAACCCGTGA |  |
| SLAPR1s | 5' CCCAAGCTTAAGAAGGTCAAAATTCAACAGCTGGGTAGCCACCTCGTCCGCCGC |  |
| SLAPR2s | 5' GGTAGCCACCTCGTCCGCCGCGTTGGATTGTTTGCCTCCCTGCTGCGGTT |  |
| SLAPFp | 5'CCCAAGCTTGCGGGAGACGTCGAGTCCAACCCTGGGCCTATGGTGAGCAAGGGCGAGGA |  |
| SLAPRp | 5' GAGCTCTTACTTGTACAGCTCGTCCATGC |  |
| pg2Am1gFp | 5' TCTAGAATGGTGAGCAAGGGCGAGG |  |
| pg2Am1gRp | 5’CCAAGCTTAAGAAGGTCAAAATTCAACAGCTGTTTCACCGGTGCCTTGTACAGCTCGTG |  |
| pg2Am2gFp | 5' TCTAGAATGGTGAGCAAGGGCGAGG |  |
| pg2Am2gRp | 5’CCAAGCTTAAGAAGGTCAAAATTCAACAGCTGCCGGGACCGGGACTTGTACAGCTCGTG |  |
| rfp-f1 | 5'- CGCGGATCCGATGGCCTCC |  |
| rfp-r1 | 5'- CCGGAATTCCTACAGGAACAGGTGG |  |
| Chl/X+B-FI1 | 5'- GCTCTAGAATGGCGCCCACCGTG |  |
| Chl/X+B-RI2 | 5 - CGGGATCCTTCCGCCGTTGCTGAC |  |
| gfp/B+K-FI3 | 5'- CGCGGATCCGGTGCATGGTGAG |  |
| gfp/B+K-RI4 | 5'- CGGGGTACCTTACTTGTACAGCTCGTCC |  |
| rfp/B+K-FI5 | 5'- CGGGATCCGGTGCATGGCCTCC |  |
| rfp/B+K-RI6 | 5'- GGGGTACCCTACAGGAACAGG |  |
| ERgfp/X+K-FII7' | 5'- CTCTAGAATGAAGACTAATCTTTTTCTCTTTCTCATCTTCTCACTTCTCCTATCATTA |  |
| ERgfp/X+K-FII7 | 5'– TTCTCCTATCATTATCCTCGGCCGAGTTCATGGTGAGCAAGG |  |
| ERgfp/X+K-RII8  (54bp) | 5'- GGGGTACCTTAAAGCTCATCATGCTTGTACAGCTCGTCCATGCCGTGAGTGATC |  |
| ERrfp/X+K-FII9' | 5'- GCTCTAGAATGAAGACTAATCTTTTTCTCTTTCTCATCTTCTCACTTCTCCTATC |  |
| ERrfp/X+K-FII9 | 5'- ATCTTCTCACTTCTCCTATCATTATCCTCGGCCGAGTTCATGGCCTCCTCCGAG |  |
| ERrfp/X+K-RII10 | 5'- GGGGTACCCTAAAGCTCATCATGCAGGAACAGGTGGTGGCGGCCCTCGGTGCGCTCGTA |  |
| gfpLP/S+HK-FV11 | 5'- TCCCCCGGGATGGTGAGCAAGGGCGAGGAGCTGTTCACCGGGGTGGTGCCCATC |  |
| gfpLP/S+HK-FV12 | 5'- ACAGCTGGGTAGCCACCTCGTCCGCCGCGTTGGACTTGTACAGCTCGTCCATG |  |
| gfpLP/S+HK-FV12' | 5'- CGGGGTACCAAGCTTAAGAAGGTCAAAATTCAACAGCTGGGTAGCCACCTCG |  |
| LPrfp/H+K-V13 | 5'- CCCAAGCTTGCGGGAGACGTCGAGTCCAACCCTGGGCCTATGGCCTCCTCCGAGAAC |  |
| LPrfp/H+K-V14 | 5'- CGGGGTACCCTACAGGAACAGGTGGTGGCGGCCCTCGGTGCGCTCGTACTGCTCCA |  |
| gfp/B+K-F | 5'- CGGGATCCATGGTGAGCAAGGGCGAG |  |
| CHgL/S+H+K-FⅢ15 | 5'- TCCCCCGGGATGGCGCCCACCGTGATGATGGCCTCGTCGGCCACCGCCGT |  |
| CHgL/S+H+K-RⅢ16 | 5'- CAACAGCTGGGTAGCCACCTCGTCCGCCGCGTTGGACTTGTACAGCTCGTC |  |
| CHgL/S+H+K-RⅢ17 | 5'- CGGGGTACCCCCAAGCTTAAGAAGGTCAAAATTCAACAGCTGGGTAGCCAC |  |
| LCHr/S+HK-FⅢ18 | 5'- CCCAAGCTTGCGGGAGACGTCGAGTCCAACCCTGGGCCTATGGCGCCCACCGTGATG |  |
| LERr/H+K-FV19 | 5'- CCCAAGCTTGCGGGAGACGTCGAGTCCAACCCTGGGCCTATGAAGACTAATCTTTTTC |  |
| ERgL/S+HK-F21 | 5'- TCCCCCGGGATGAAGACTAATCTTTTTCTCTTTCTCATCTTCTCACTTCTCCTATC |  |
| ERgL/S+HK-R22 | 5'- AACAGCTGGGTAGCCACCTCGTCCGCCGCGTTGGAAAGCTCATCATGCTTGTACAG |  |
| ERgL/S+HK | 5'- CGGGGTACCCCCAAGCTTAAGAAGGTCAAAATTCAACAGCTGGGTAGCCACC |  |
| ERrfp/s+k-FV15 | 5'- TCCCCCGGGATGAAGACTAATCTTTTTCTCTTTCTCATCTTCTCACTTCTCCTATC |  |
| ERgfp/s+k-FV17 | 5'- TCCCCCGGGATGAAGACTAATCTTTTTCTCTTTCTCATCTTCTCACTTCTCC |  |

Reference:

1.Gnanasambandam A, Polkinghorne IG, Birch RG （2007）Heterologous signals allow efficient targeting of a nuclear-encoded fusion protein to plastids and endoplasmic reticulum in diverse plant species. Plant Biotechnol J 5: 290～2962.

2.Lebrun M, Waksman G, Freyssinet G (1987) Nucleotide sequence of a gene encoding corn ribulose-1,5-bisphosphate carboxylase/oxygenase small subunit (rbcs). Nucleic Acids Res 15:4360
